# Supplementary material for: Activin E is a transforming growth factor β ligand that signals specifically through activin receptor-like kinase 7
Source: Biochem J. 2024 Apr 10;481(7):547–64. doi: 10.1042/BCJ20230404 (PMC11088876; doi:10.1042/BCJ20230404)
Supplement: Supplementary Material [file BCJ-481-547-s1.pdf]

**Supplementary Table 1.** PDBePISA analysis of the type II interface (ActRIIB) of activin ternary models.

| **Type II (ActRIIB)** | | |
|---|---|---|
| **Solvent-accessible (Å$^2$)** | | |
| **Ligands** | **Interface** | **Total** |
| **ActB** | 738 | 7641 |
| **ActC** | 779 | 7635 |
| **ActE** | 751 | 7408 |
| **ActA (PDB: 7OLY)** | 680 | 7726 |

**Supplementary Table 2.** PDBePISA analysis of the fingertip region of the type I interface (ALK4 or ALK7) of activin ternary models.

| **Type I Fingertips (Chain A)** | | |
|---|---|---|
| **Solvent-accessible (Å$^2$)** | | |
| **Ligands** | **Interface** | **Total** |
| **ActB** | 401 | 7651 |
| **ActC** | 447 | 7635 |
| **ActE** | 375 | 7408 |
| **ActA (PDB: 7OLY)** | 346 | 7726 |

**Supplementary Table 3.** PDBePISA analysis of the prehelix/wrist region of the type I interface (ALK4 or ALK7) of activin ternary models.

| **Type I Prehelix/wrist (Chain B)** | | |
|---|---|---|
| **Solvent-accessible (Å$^2$)** | | |
| **Ligands** | **Interface** | **Total** |
| **ActB** | 785 | 7641 |
| **ActC** | 837 | 7599 |
| **ActE** | 781 | 7386 |
| **ActA (PDB: 7OLY)** | 617 | 7726 |

**Supplementary Table 4.** Root mean square difference (RMSD) measurements of the fingertip regions of the type I interfaces.

| **Fingertip Region (Chain A)** | | | |
|---|---|---|---|
| | **ActB** | **ActC** | **ActE** |
| **ActB** | 0 | 0.19 | 0.231 |
| **ActC** | 0.19 | 0 | 0.212 |
| **ActE** | 0.231 | 0.212 | 0 |
| **ActA (PDB: 7OLY)** | 0.364 | 0.401 | 0.396 |

**Supplementary Table 5.** Root mean square difference (RMSD) measurements of the prehelix/wrist regions of the type I interfaces.

| **Prehelix/Wrist Region (Chain B)** |
|---|

| | ActB | ActC | ActE |
|---|---|---|---|
| **ActB** | 0 | 0.86 | 0.808 |
| **ActC** | 0.86 | 0 | 0.195 |
| **ActE** | 0.808 | 0.195 | 0 |
| **ActA (PDB: 7OLY)** | 1.047 | 0.659 | 0.761 |

**Supplementary Table 6.** PDBePISA analysis of the type II interface (ActRIIA) of activin ternary models.

| **Type II (ActRIIB)** | | |
|---|---|---|
| | **Solvent-accessible (Å²)** | |
| **Ligands** | **Interface** | **Total** |
| **ActB** | 749 | 7785 |
| **ActC** | 755 | 7729 |
| **ActE** | 752 | 7492 |
| **ActA** | 775 | 7711 |

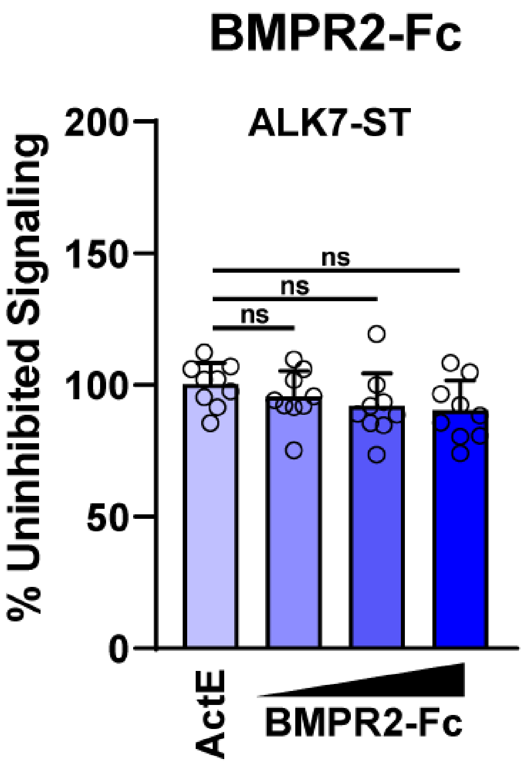


**Supplemental Figure 1. Activin E signaling does not require the type II receptor, BMPR2, to signal.** ($CAGA_{12}$)-luciferase HEK293T cells transfected with ALK7-ST receptor constructs and treated with ActE conditioned media (20x), and 10μM SB-431542 in the presence of increasing amounts (6.25, 12.5, 25 nM) of BMPR2-Fc (ns = not significant).

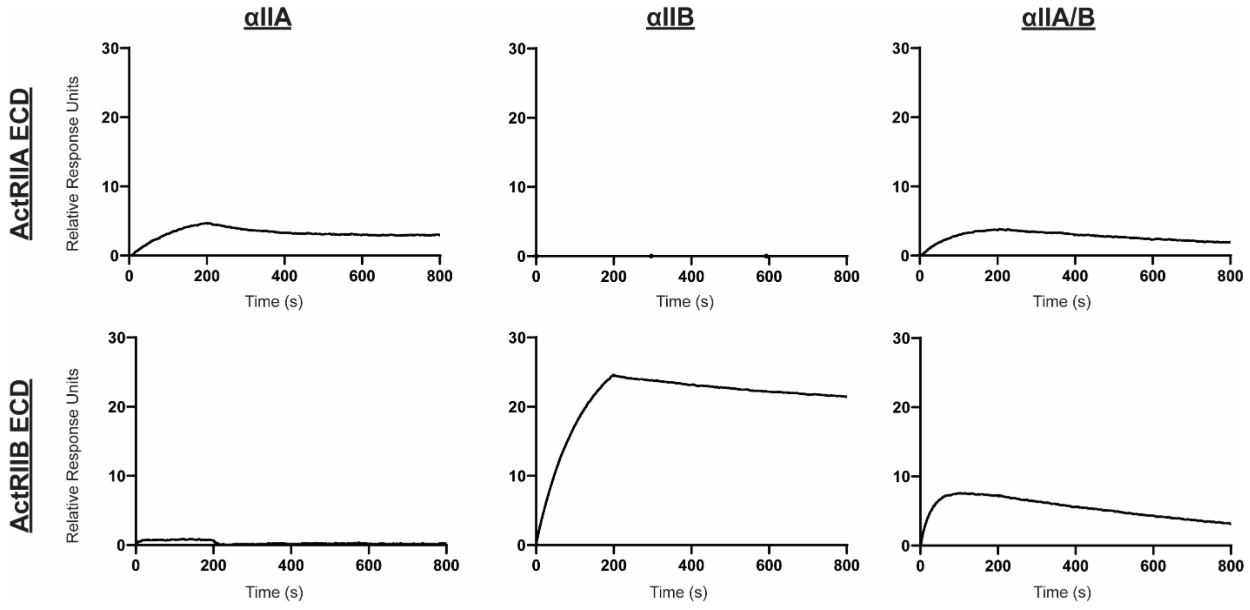


**Supplemental Figure 2. Binding specificity of the activin type II neutralizing antibodies.** Representative surface plasmon resonance (SPR) sensorgrams of the ActRIIA ECD (25nM) or ActRIIB ECD (25nM) binding to protein A captured anti-ActRIIA nAb, anti-ActRIIB nAb, or anti-ActRIIA/ActRIIB nAb.

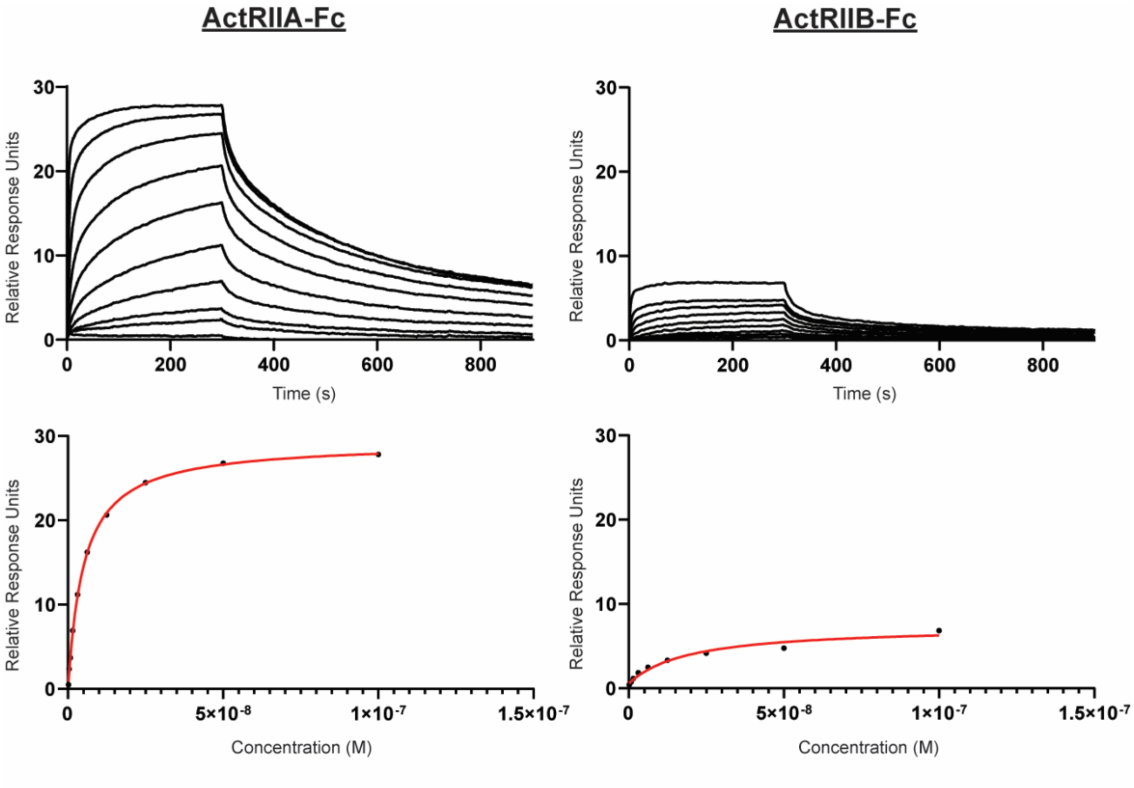


| Receptor | KD (M) | Chi² (RU²) |
|---|---|---|
| ActRIIA-Fc | 5.0E-09 | 0.066 |
| ActRIIB-Fc | 2.0E-08 | 0.17 |

**Supplemental Figure 3. Activins E binds to the activin type II receptors with low affinity.** Representative surface plasmon resonance (SPR) sensorgrams of ActE (R&D Systems) binding to ActRIIA-Fc or ActRIIB-Fc capture on a protein A sensor chip. The top represents the unfit SPR curves while the bottom depicts the steady analysis of the top experiments.

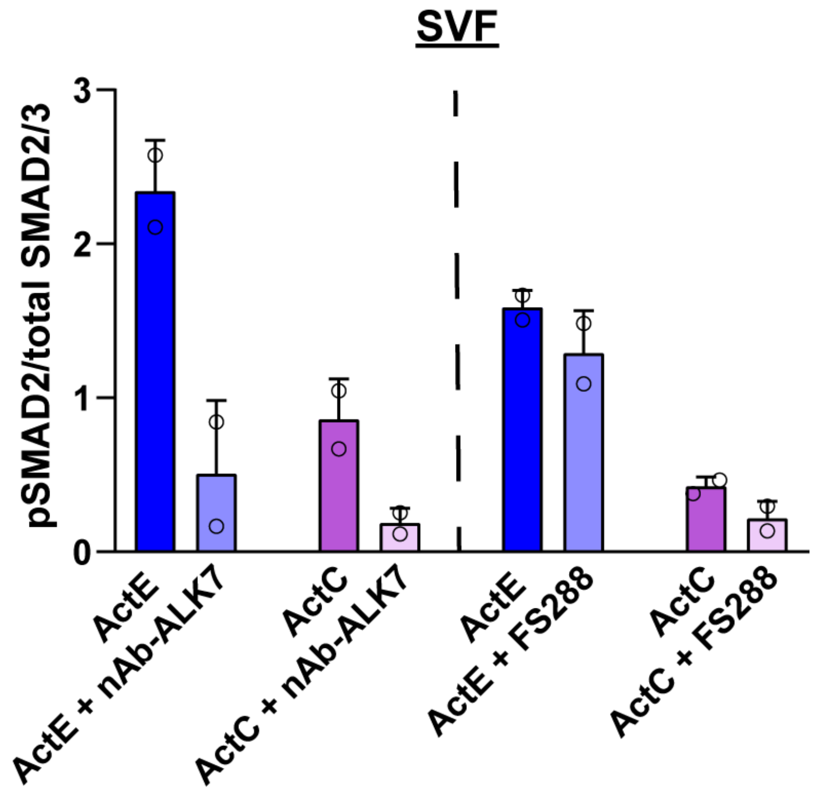


**Supplementary Figure 4. Quantitation of SVF western blot images.** Quantitative analysis of western blot images in used in Figure 7 for changes in signaling following treatment with either ActE conditioned media (20x) or ActC in the presence or absence of an ALK7 neutralizing antibody or FS 288. Images were quantified using ImageJ software.

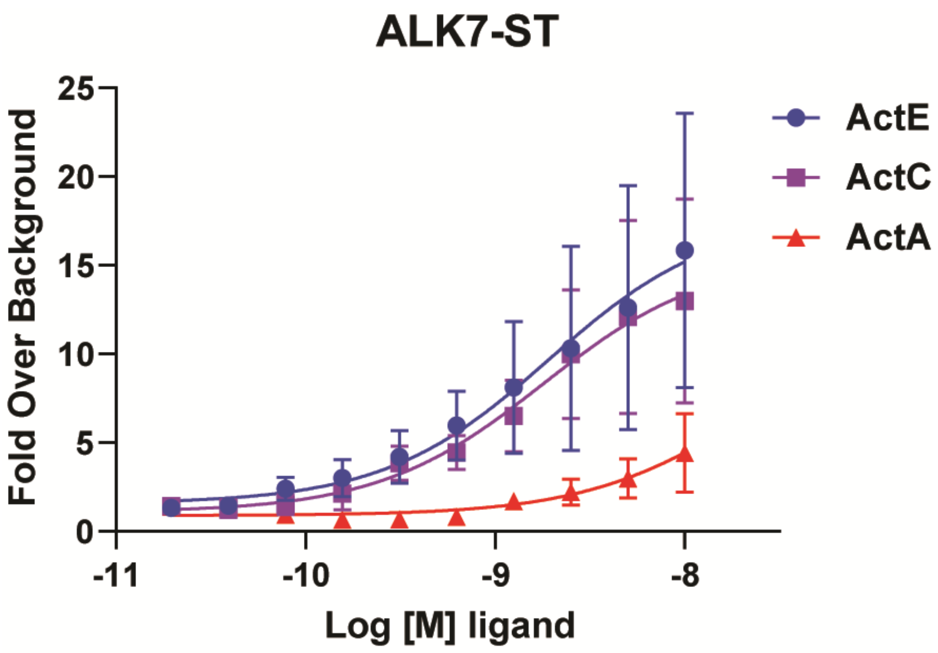


| Ligand | EC50 (M) | SD | 95% CI |
|---|---|---|---|
| ActE | 2.0E-09 | 1.6E-10 | 7.8e-010 to 5.3e-009 |
| ActC | 1.6E-09 | 8.9E-10 | 9.5e-010 to 3.5e-009 |
| ActA | NA | NA | NA |

**Supplemental Figure 5. Recombinant ActE signals through ALK7.** Titration of recombinant mature ActA, ActC, or ActE from R&D Systems (10 nM) in (CAGA$_{12}$)-luciferase HEK293T cells transfected with SB-431542 resistant ALK7-ST. EC50 values were calculated using non-linear dose response curve fitting in Prizm. The ActA curve was not fit to a non-linear regression (NA=Not Applicable).

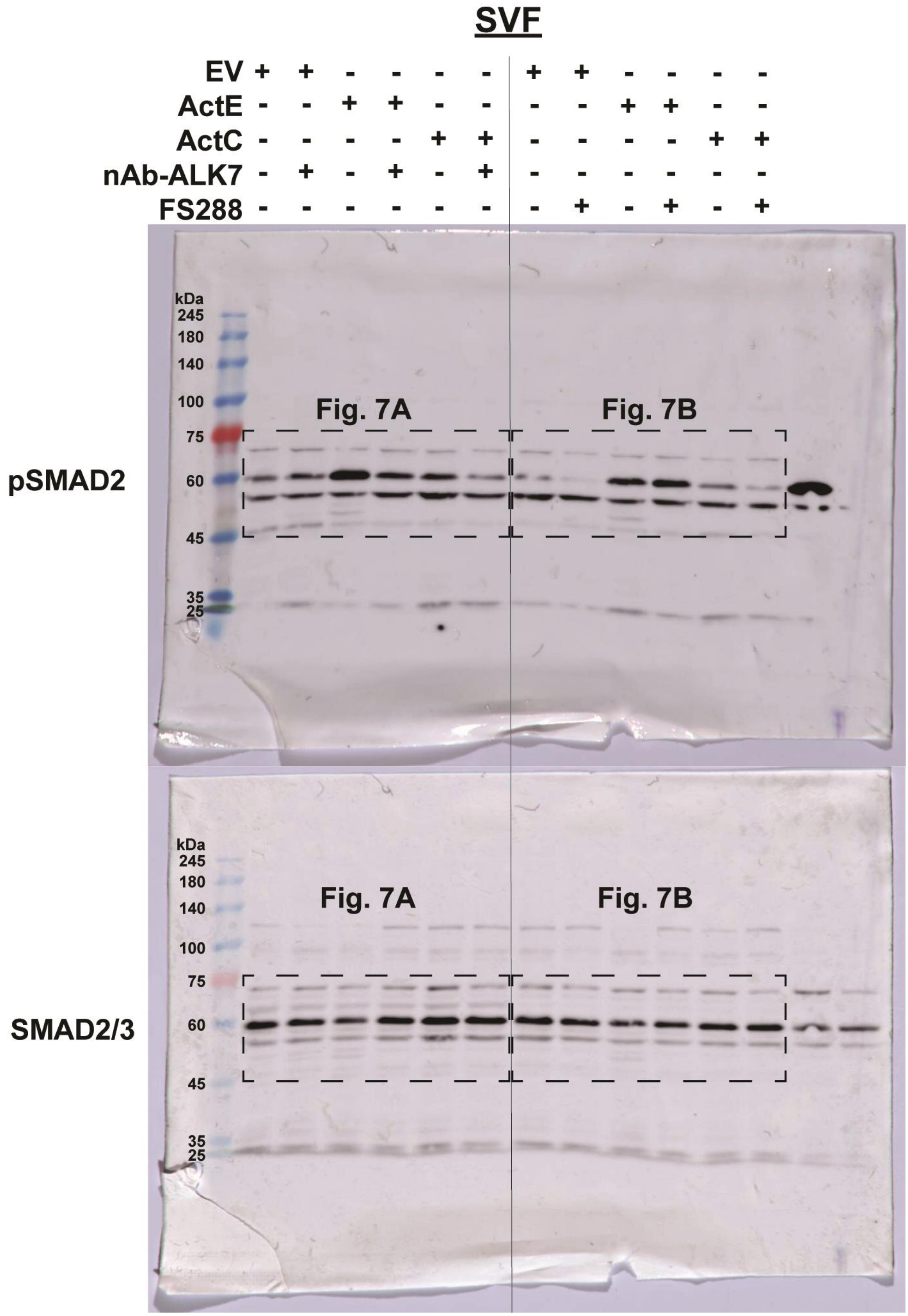
SVF
EV + + - - - - + + - - - -
ActE - - + + - - - - + + - -
ActC - - - - + + - - - - + +
nAb-ALK7 - + - + - + - - - - - -
FS288 - - - - - - - + - + - +
kDa
245
180
140
100
75
60
45
35
25
Fig. 7A
Fig. 7B
pSMAD2
SMAD2/3

**Supplemental Figure 6. Raw pSMAD2 and SMAD2/3 western blots images.** Supplemental western blot images for blots shown in Figure 7. Dashed boxes depict where the images were cropped for figure generation.
